# Supplementary material for: Snail-inspired robotic swarms: a hybrid connector drives collective adaptation in unstructured outdoor environments
Source: Nat Commun. 2024 Apr 29;15:3647. doi: 10.1038/s41467-024-47788-2 (PMC11058817; doi:10.1038/s41467-024-47788-2)
Supplement: Supplementary file 1 — Supplementary information [file 41467_2024_47788_MOESM1_ESM.pdf]

Supplementary Information for Snail-inspired robotic swarms: a hybrid connector drives collective adaptation in unstructured outdoor environments

Da Zhao[1,2], Haobo Luo[1,2], Yuxiao Tu[1,2], Chongxi Meng[1,2], Tin Lun Lam[1,2*]

[1]School of Science and Engineering,
The Chinese University of Hong Kong, Shenzhen, China.
[2]Shenzhen Institute of Artificial Intelligence and Robotics for Society, Shenzhen, China.
*Corresponding author. Email: tllam@cuhk.edu.cn

This PDF file includes:

Supplementary Note 1. Geometric parameter optimization.

Supplementary Note 2. Optimization of magnetic and driving Forces.

Supplementary Note 3. Bending force conversion.

Supplementary Note 4. A simplified steering model for torque analysis.

Supplementary Note 5. Comparison with other modular self-reconfigurable robots.

Supplementary Tables 1 to 4, Supplementary Figures 1 to 22.

Other Supplementary Information for this manuscript includes the following:

Supplementary Movies 1: Overview of the proposed snail robot swarm.

Supplementary Movies 2: Bioinspired dual-mode connection mechanism deisgn.

Supplementary Movies 3: Individual robot's locomotion ability.

Supplementary Movies 4: Collaborate to climb a rock step.

Supplementary Movies 5: Traverse a cobblestone road.

Supplementary Movies 6: Collaborate to build a bridge to cross the gully.

Supplementary Movies 7: Robotic manipulation.

Supplementary Note 1. Geometric parameter optimization

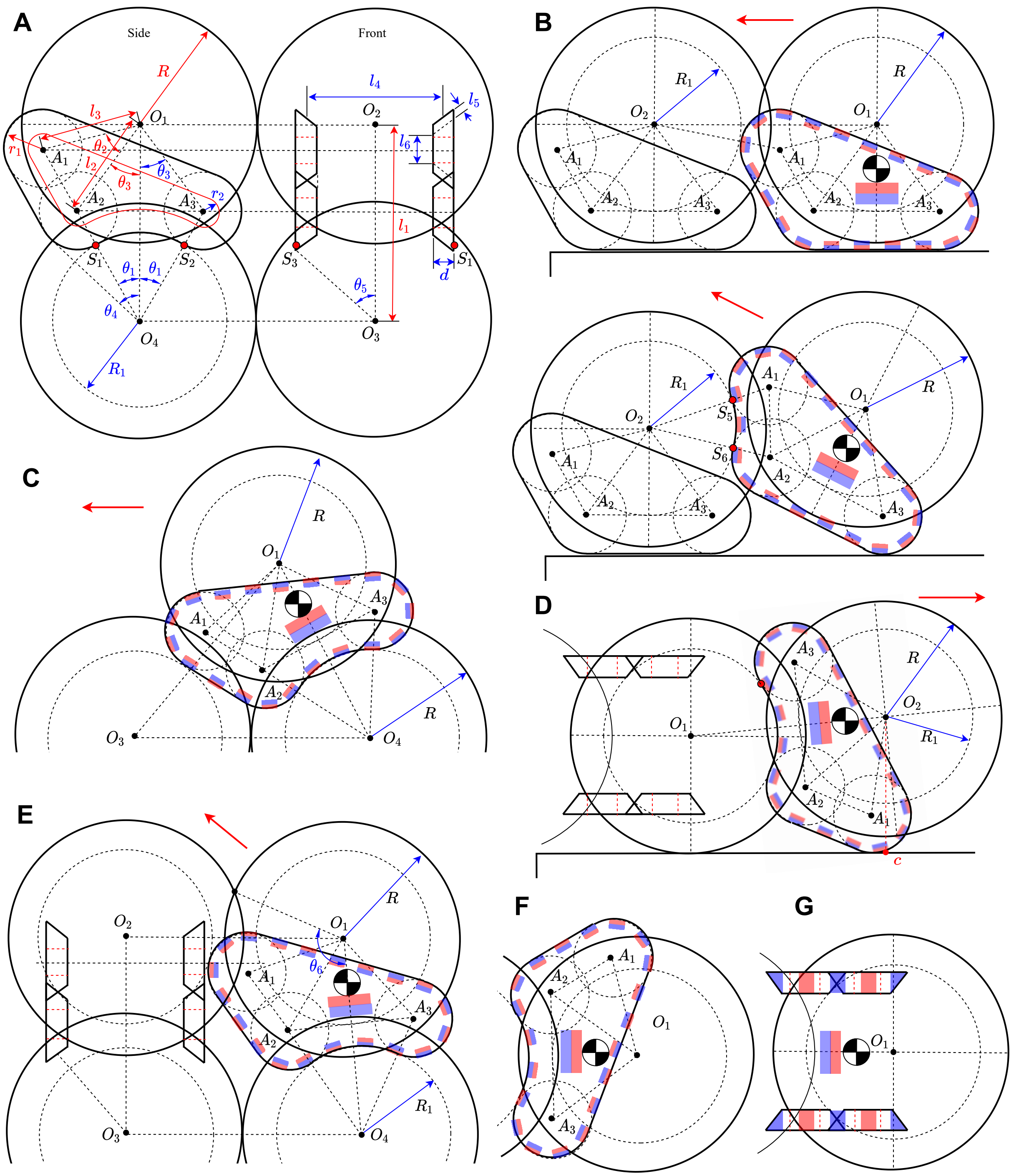

Supplementary Fig. 1. Symbolic representation of the snail robot and constraints of self-reconfiguration. (**A**) Symbolic representation of dimension parameters. (**B**) The robot tries to attach to its peers. (**C**) The robot tries to execute the adjacent transition. (**D**) The robot prepares to separate from its peers. (**E**) The robot prepares to execute the nonadjacent transition action. (**F**) & (**G**): Two cases of cantilever structure.

Geometric constraints

When in free mode, a snail robot has four basic actions: connection, separation, adjacent transition, and non-adjacent transition. To successfully perform these basic actions, there are many geometric and force constraints on the snail robot, as shown in Supplementary Fig. 1. We symbolically represent the snail robot when it connects to other peers, as shown in Supplementary Fig. 1A. After some simple analysis, we know that there are at least 7 dimension parameters to define a robot, which are R, θ_2, θ_3, l_1, l_2, l_3, r_1. Their symbols are shown in red in Supplementary Fig. 1A and other parameters can be calculated based on these seven red ones.

The calculation progress of other parameters that are shown in blue:

$$l_{A_2O_4} = \sqrt{l_2^2 + l_1^2 - 2l_1l_2\cos(\theta_3)} \tag{1}$$

$$R_1 = l_{A_2O_4} - r_1 \tag{2}$$

$$l_4 + d = 2\sqrt{R^2 - R_1^2} \tag{3}$$

$$l_{A_1A_2} = \sqrt{l_3^2 + l_2^2 - 2l_3l_2\cos(\theta_2)} \tag{4}$$

$$l_{A_2A_3} = \sqrt{l_2^2 + l_2^2 - 2l_2l_2\cos(2\theta_3)} \tag{5}$$

$$l_{A_1O_4} = \sqrt{l_3^2 + l_1^2 - 2l_1l_3\cos(\theta_2 + \theta_3)} \tag{6}$$

$$l_6 = r_1 - 2(d\tan\theta_5 + \tfrac{l_5}{\cos\theta_5}) \tag{7}$$

d is the width of the belt, which is a constant:

$$d = constant_1 \tag{8}$$

To ensure the snail robot can connect to at least 5 robots simultaneously, the coverage area of four guide wheels needs to be limited. In Supplementary Fig. 1A, the angle θ_4 and θ_5 should be less than $\pi/4$, resulting in the following condition:

$$\theta_4 = \arcsin\frac{r_1}{R_1 + r_1} + \arcsin\frac{l_2 \sin\theta_3}{R_1 + r_1} < \frac{\pi}{4} \tag{9}$$

$$\theta_5 = \arcsin\frac{l_4 + d}{\sqrt{(l_4 + d)^2 + 4(l_1 - l_2 cos\theta_3 - r_1)^2}} \leq \frac{\pi}{4} \tag{10}$$

The position of the front wheel A_1 should also be limited to prevent collisions with other modules. Then we have the following:

$$\angle O_1O_4A_1 = \arcsin\frac{l_3\cos(\theta_2 + \theta_3)}{l_{A_1O_4}} \tag{11}$$

$$\arcsin\frac{r_1}{l_{A_1O_4}} + \angle O_1O_4A_1 \leq \frac{\pi}{4} \tag{12}$$

In order to add a sucker at the bottom of the robot, $l_4 - d$ must be restricted, then we have

$$l_4 - d > constant_3 \tag{13}$$

We can also add this equation to our objective function, to maximize it.

$$\begin{aligned} l_4 + d &= 2\sqrt{R^2 - R_1^2} \\ &= 2\sqrt{R^2 - (\sqrt{l_1^2 + l_2^2 - 2l_1l_2\cos\theta_3} - r_1)^2} \end{aligned} \tag{14}$$

The geometric parameter representation of the connection process is shown in Supplementary Fig. 1B. In fact, due to Eq. 12, it is very difficult for wheel A1 to touch the shell of the front module when the two shells are tangent. The same happens when the robot performs a transition action or a non-adjacent transition action, as shown in Supplementary Figs. 1C and 1E. So, part of the front of the shell needs to be cut off to prevent a collision. In this case, there is no strict constraint on the geometry of the robot when performing these three actions.

However, we can set shell-cutting reduction as one of the optimization goals to increase the connection region. In Supplementary Fig. 1, we know that cutting the most shell occurs when the robot performs the nonadjacent transition. So, θ_6 should be minimized. In Supplementary Fig. 1E, θ_6 is given by

$$\theta_6 = \theta_2 + \theta_3 + \arccos \frac{l_{O_1 O_2}}{2R} \tag{15}$$

In Supplementary Fig. 1A, S_1, S_2, S_3, S_4 denote the four edge support points of the snail robot when it connects to another module. If the distance between these support points is too small, the robot is susceptible to tipping over due to its own weight, as illustrated in Supplementary Figs. 1F and 1G. Therefore, the distances between these supports need to be as large as possible, while also satisfying other constraints.

In Supplementary Fig. 1A, l_5 represents the thickness of the track magnet. l_6 is the rotation diameter of the synchronous belt inside the track, which is given by

$$l_6 = r_1 - 2(d \tan \theta_5 + \frac{l_5}{\cos \theta_5}) \tag{16}$$

l_6 can not be too small, otherwise, the track will be subjected to greater bending stress at the regulating wheel, affecting its service life. We can add a constraint that makes it greater than a constant.

In Supplementary Fig. 1D, when the snail robot tries to detach from another one, its front wheels need to touch the ground to provide a friction force for a forward drive. Then we have a constraint:

$$O_2 c > R \tag{17}$$

Nonlinear Optimization

The geometric parameter optimization problem of this modular robot in Free mode can be formulated as a nonlinear optimization problem. We set the reduction of the shell cutting and

the increase in the distance between support points as the optimization object, and the geometric constraints mentioned above as the nonlinear constraints. Therefore, this optimization problem can be formulated as

$$
\begin{aligned}
&\min \;\; f = w_1\theta_6 + w_2(-l_{S_1S_2}) + w_3(-l_{S_3S_1}) \\
&s.t. \begin{cases}
\theta_4, \theta_5 \leq \frac{\pi}{4} \\
\arcsin \frac{r_1}{l_{A_1O_4}} + \arcsin \frac{l_3 \cos(\theta_2+\theta_3)}{l_{A_1O_4}} \leq \frac{\pi}{4} \\
O_1c > R \\
l_6 \geq constant_4 \\
for\ x_i, (i = 1, 2 \cdots 6) : lb_i \leq x_i \leq ub_i.
\end{cases}
\end{aligned}
$$

where x_i, $(i = 1, 2 \cdots 6)$ represent the six basic sizes $R, \theta_2, \theta_3, l_1, l_2, l_3, r_1$. lb_i and ub_i are their lower and upper bounds. w_1, w_2, w_3 are the weight coefficients.

With the help of some nonlinear optimization solvers, we can get suitable parameter values. For example, if we set the radius of the shell R to 120mm, then we can get r_1^*=20mm, θ_2^*=41.95deg, θ_3=36.16deg, l_1^*=100.04mm, l_2^*=51.35mm, l_3^*=50.85mm.

Supplementary Note 2. Optimization of Magnetic and Driving Forces

This section will focus on the optimization of the magnetic and driving forces of the snail robot. The optimization mentioned above in Supplementary Note 1 is about geometry. In fact, there are also many magnetic force constraints for snail robots. The reason for considering them separately is that geometric optimization aims to maximize the region of support points, which must be beneficial to the robot's connection strength. Additionally, the bigger region of support points must benefit the resistance to bending forces in strong mode.

The Calculation progress of tipping over risk ratio and slipping Risk Ratio

Tipping Over Risk Ratio (TORR) denotes the likelihood of the robot tipping over in its current state; a value approaching 1 implies a higher probability of tipping over. The slipping Risk Ratio (SPRR), expresses the risk of the robot slipping in its present state; a value closer to 1 indicates a greater likelihood of slipping.

As shown in Supplementary Fig. 2, we have depicted several individual robots performing circular motions on the surface of a spherical shell, where P is the center of the mass. The relationship between the center of gravity position of the robot and the position of the chassis support points directly affects TORR. When the robot's center of gravity is within the support region of the supporting points (S_1 and S_2) in the vertical direction, TORR remains 0. We denote the angle between O_1O_2 and the vertical line as γ. When COM and the supporting point are vertically aligned, we denote the angle γ as γ^*. Therefore, we can conclude that when $-\gamma^* < \gamma < \gamma^*$, TORR = 0. This means that if the projection of COM in the vertical direction falls within the support area of the robot's chassis, tipping over is impossible. As γ gradually increases, TORR also starts to increase. The distance between COM and the supporting point in the horizontal direction as L, which is given by

$$L = \begin{cases} l_{PS_1} \left|\sin(\gamma - \gamma^*)\right|, & \gamma^* \le \gamma \le \pi \\ l_{PS_1} \left|\sin(\gamma + \gamma^*)\right|, & \pi < \gamma \le 2\pi - \gamma^* \end{cases} \tag{18}$$

Once we obtain L, it is easy to derive the expression for TORR of a snail robot moving on the spherical shell, which is written as follows:

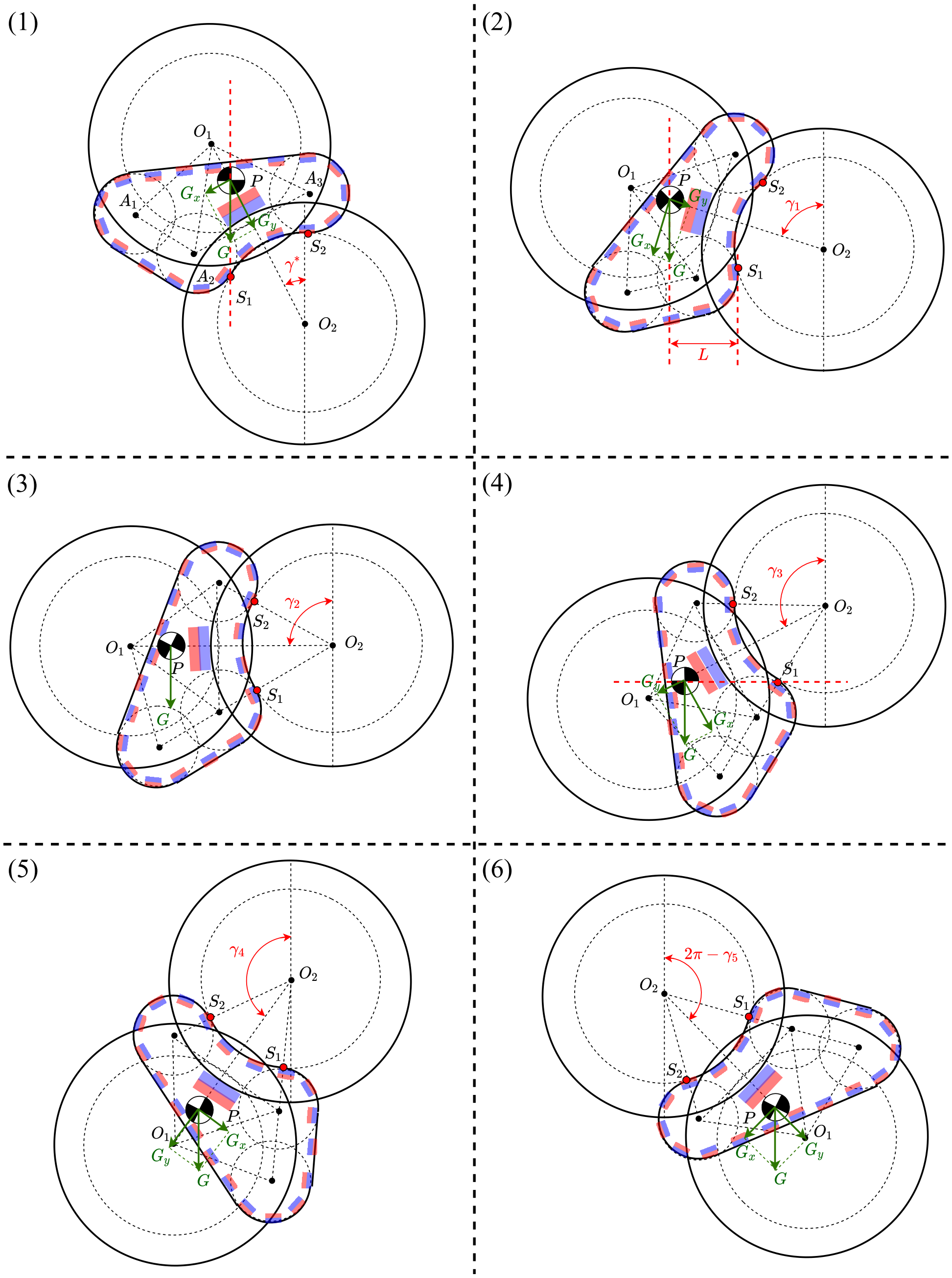

Supplementary Fig. 2. Several scenarios of snail robot moving on another spherical shell.

$$TORR = \frac{2GL}{l_{S1S2}(F_n)_{max}} \tag{19}$$

For the more general case of calculating TORR, the main consideration is whether the pro-

jection of the robot's center of gravity is within the support points. If it is inside the support points, TORR is 0. If it is not inside the support points, then the TORR is calculated as the ratio between the moment caused by the center of gravity with respect to the static equilibrium point and the moment caused by the magnetic forces applied to the robot with respect to the static equilibrium point.

As depicted in Supplementary Fig. 2, SPRR is primarily caused by the gravitational components of the robot, which is given by

$$SPRR = \max(\frac{(G - G_y)\mu + G_x, 0}{(F_S)_{max}}, 0) \tag{20}$$

where

$$G_x = G\,|\sin\gamma| \tag{21}$$

$$G_y = G\cos\gamma \tag{22}$$

μ is the friction coefficient between the track and the spherical shell.

For the more general case of calculating SPRR, we need to measure the maximum forward force that the robot can provide before slipping occurs at a particular position. Then, SPRR is calculated as 1 minus the ratio between this measured maximum forward force and the robot's maximum resistance to lateral forces.

The Calculation Progress of Resistance Risk Ratio

Resistance Risk Ratio (RERR) represents the proportion of the robot's experienced resistance to its driving force, which is given by the ratio of the forward resistance to its maximum driving force.

When running on the ground, the robot's maximum driving force is given by

$$F_{d1} = \begin{cases} G\mu_1, & \text{if } \eta\frac{i\tau_m}{r_1} > G\mu_1 \\ \eta\frac{i\tau_m}{r_1}, & \text{if } \eta\frac{i\tau_m}{r_1} < G\mu_1 \end{cases} \tag{23}$$

where η is the transmission efficiency between the motor and the track, including the gear drive. μ_1 is the friction coefficient between the track and the ground. τ_m is the output torque of the driving motor. i is the transmission ratio between the motor and the track drive wheel. The relationship between the maximum forward driving force and μ_1, when the snail robot moves on the ground, is illustrated in Supplementary Fig. 3.

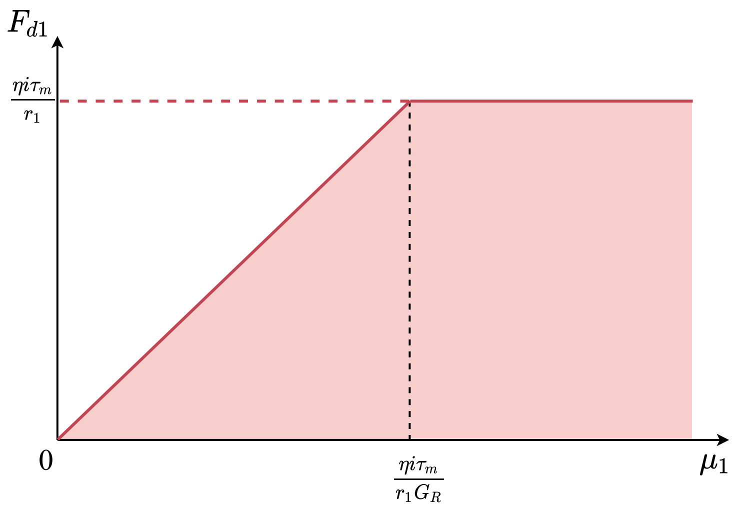

Supplementary Fig. 3. The relationship between the maximum forward driving force and μ_1 when snail robot moves on the ground.

When running on other module's shell, the maximum driving force is given by

$$F_{d2} = \begin{cases} F_R \mu, & \text{if } \eta \dfrac{i\tau_m}{r_1 - d\tan\theta_5} > F_R \mu \\ \eta \dfrac{i\tau_m}{r_1 - d\tan\theta_5}, & \text{if } \eta \dfrac{i\tau_m}{r_1 - d\tan\theta_5} < F_R \mu \end{cases} \tag{24}$$

F_R represents the supporting force of the spherical shell on the robot, which is given by

$$F_R = \begin{cases} F_M + G\cos\gamma, 0 \leq \gamma \leq \gamma^* \ or \ 2\pi - \gamma^* \leq \gamma \leq 2\pi \\ F_M + G\cos\gamma - \dfrac{2G}{l_{S1S2}} l_{PS_1} \left|\sin(\gamma - \gamma^*)\right|, \gamma^* \leq \gamma \leq \pi \\ F_M + G\cos\gamma - \dfrac{2G}{l_{S1S2} l_{PS_1}} \left|\sin(\gamma + \gamma^*)\right|, \pi \leq \gamma \leq 2\pi - \gamma \end{cases} \tag{25}$$

where F_M is the attraction force of the robot's magnets to another robot's spherical shell.

Then we can obtain the calculation formula for RERR when the snail robot moves on another spherical shell:

$$RERR = \frac{\max(F_m \pm G\sin\gamma, 0)}{F_{d2}} \tag{26}$$

Where the plus-minus sign is negative when the direction of the robot's movement is the same as the direction of the gravitational component G_x; otherwise, it is positive. F_m is the resistance of magnets on the caterpillar to the robot's forward movement.

For the more general case of calculating RERR, we need to measure the maximum forward force that the robot can provide before it is unable to move forward at a particular position. Then, RERR is calculated as 1 minus the ratio between this measured maximum forward force and the robot's maximum forward driving force.

Finally, we obtain the calculation methods for three risk rates when the robot moves on another iron spherical shell. By substituting the corresponding parameters, we can obtain the curves of the three risk ratios as γ varies from 0 to 2π, as shown in Supplementary Fig. 4. From the figure, we can derive some evident information. For instance, the curves of TORR and SPRR are symmetric concerning the axis $\gamma = \pi$. When $\gamma = \frac{\pi}{2} + \gamma^*$, as shown in Supplementary Fig. 2(4), TORR reaches its maximum. When $\gamma = \frac{\pi}{2}$, RERR reaches its minimum, and when $\gamma = \frac{3\pi}{2}$, RERR reaches its maximum. Throughout the entire process, the risk rate of the robot experiencing slippage is relatively small. It is worth noting that the snail robot has several self-reconfiguration motions, not only the fully connected state on the spherical shell but also many intermediate states. However, the calculation process for many of these cases is quite complex. We only perform calculations for certain key positions and then fit the curves.

Magnetic and driving forces optimization

The robot has two connecting surfaces, which are formed by regulating wheels A_1, A_2 and regulating wheels $A2$, $A3$, respectively. As shown in the lower part of Supplementary Fig. 1B, when the connecting surface is the one formed by A_1 and A_2, the support points are S_5 and S_6, which are too close. Therefore, it is nearly impossible for the robot to be stable on this connecting surface. Another reason for low detach force for the track embedded with magnets

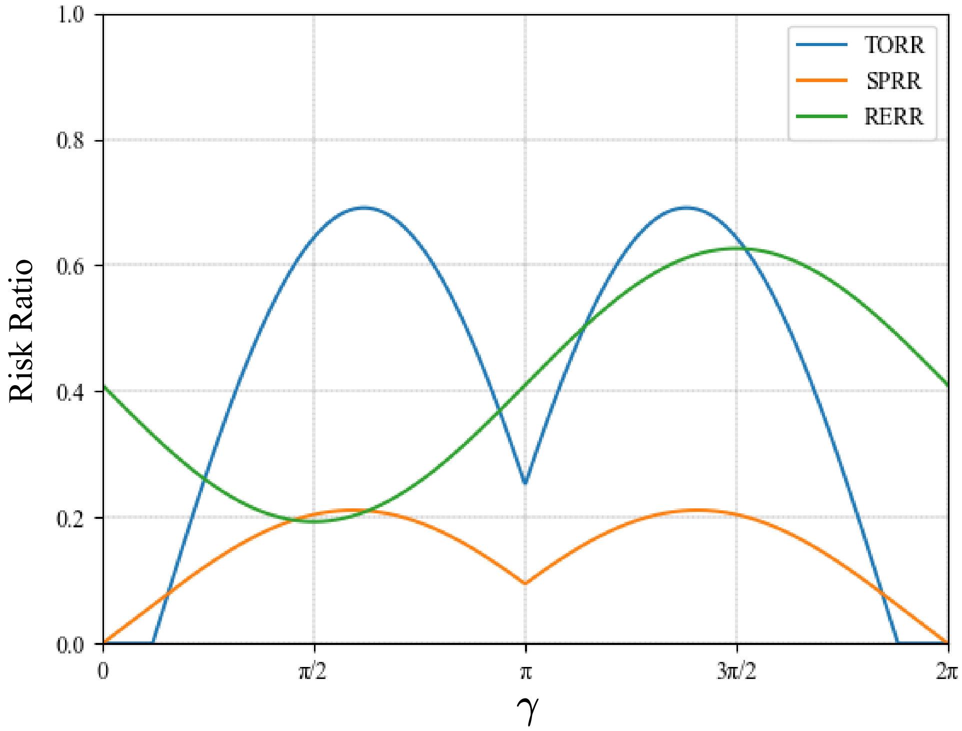

Supplementary Fig. 4. Curves of three risk ratios when snail robot moves on another iron spherical shell.

is that it has the peel-off effect, as shown in Supplementary Fig. 20. The track can be detached by a small force because of this effect. So we add some additional magnets at the bottom of the robot, which is clearly shown in Fig. 3b.

For snail robots, the magnetic force must be neither too strong nor too weak. Sufficient connection force benefits connecting more modules, but it will also cause difficulties for self-reconfiguration actions. The robot will not be able to detach from the module it attaches to if the magnetic force is too large. This situation usually occurs in separation, adjacent transition, and nonadjacent transition actions, as shown in Supplementary Figs. 1C, 1D and 1E. In these cases, it is often challenging to simultaneously control the three risk rates mentioned earlier to be below 1, especially RERR and TORR, as conflicts may frequently arise. The selection and optimization of magnetic force and driving force need to be considered simultaneously. The final specifications of the robot and the magnets used can be found in Supplementary Tables 2 and 3, respectively.

Supplementary Note 3. Bending force conversion

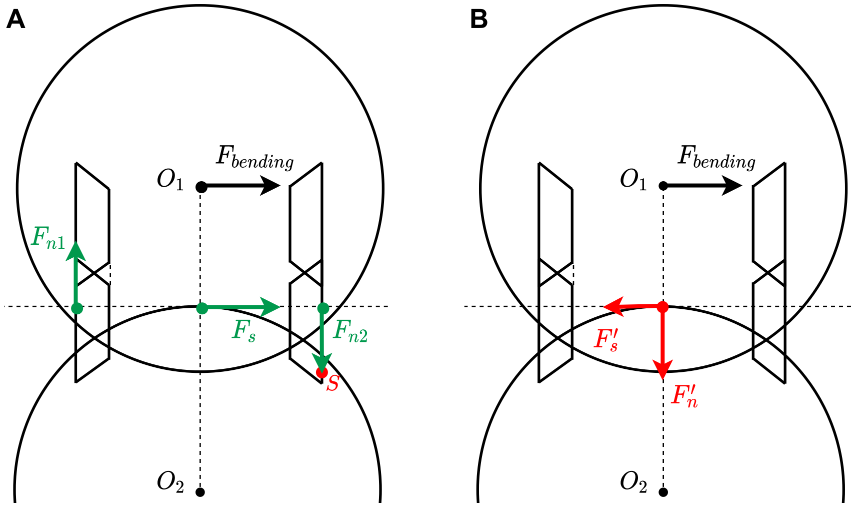

Supplementary Fig. 5. The bending force applied to the robot is converted into shear force and normal force applied to the intermediate connector.

As shown in Supplementary Fig. 5A, if the point of application of the bending force exerted on the center of the shell is transferred to the center of the bottom, it will first become a shear force F_s and a moment of couple F_n, which are shown in green. Their magnitudes are given by

$$\begin{cases} F_s = F_{bending} \\ F_n = \dfrac{F_{bending}(l_1 - R)}{l_4 + d} \end{cases} \tag{27}$$

As shown in Supplementary Fig. 5B, to resist the tangential force F_s, the intermediate connector needs to provide a tangential force F_s' in the opposite direction to F_s. At the same time, to resist the moment force F_{n1}, the intermediate connector needs to provide a normal force F_n'.

In summary, the robot's connector strength enhancement mechanism only needs to provide tangential force and normal force to resist the bending force applied to the robot.

Supplementary Note 4. A simplified steering model for torque analysis

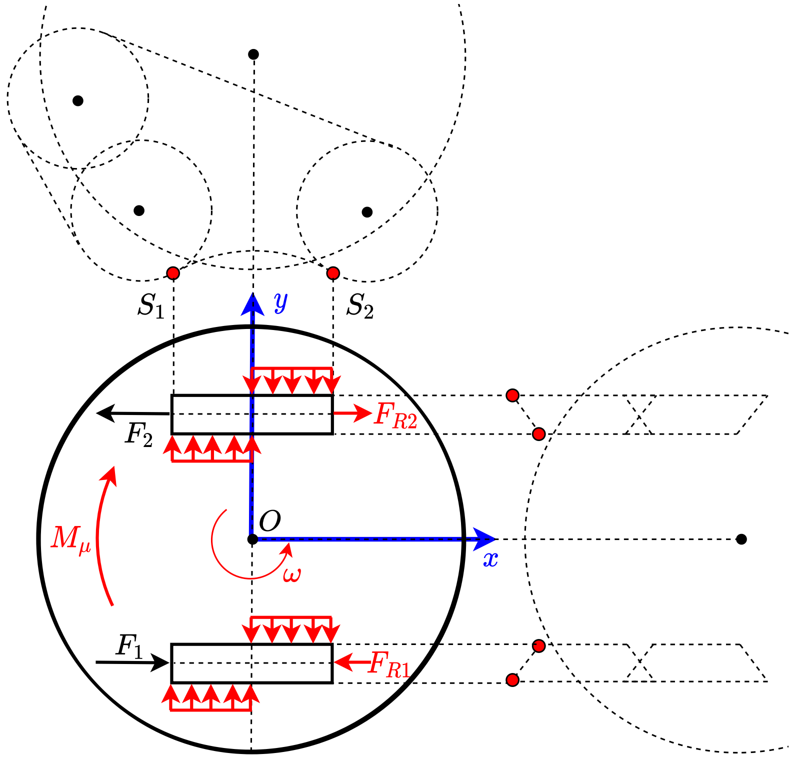

Supplementary Fig. 6. Steering model for snail robot's chassis.

In this section, we try to find the relationship between the tracks' driving forces, the resistance on the track, and the robot's output steering torque. As shown in Supplementary Fig. 6, when a robot is connected to the shell of another robot, only the tracks from S_1 to S_2 of both robots make contact. The rotation center is O. ω is the turning speed of the robot. F_1 and F_2 are the tractions of the tracks. F_{R1} and F_{R2} are the resistances of the spherical shell to the tracks, which are mainly caused by the resistance of the magnets disengaging from the spherical shell. M_μ is the steering resistance moment applied on the tracks. Then we can have

$$M_O = (F_2 + F_1)\frac{l_4}{2} - (F_{R2} + F_{R1})\frac{l_4}{2} - M_\mu + M_s \tag{28}$$

where M_O is the steering torque of the robot along the z axis and M_s is the rotation torque of the sucker. When the snail robot is in free mode, M_s is equal to 0.

We use F_{q1} and F_{q2} to represent the lateral drag load per unit length of the track. When the rotating robot is vertically placed above the spherical shell of another robot, F_{q1} and F_{q2} are as follows:

$$F_{q1} = F_{q2} = \frac{G + F_M}{2R_1\theta_1} \tag{29}$$

The steering resistance moment M_μ is given by

$$M_\mu = \mu \,\mathrm{sgn}(\theta) \int_{-\theta_1}^{\theta_1} \left(F_{q1} + F_{q2}\right) \theta R_1 \,\mathrm{d}\theta = \mu R_1 \left(F_{q1} + F_{q2}\right) \theta_1^2 \tag{30}$$

where $\mathrm{sgn}(\theta)$ is defined as

$$\mathrm{sgn}(\theta) = \begin{cases} +1, \text{ if } \theta > 0 \\ \ \ 0, \text{ if } \theta = 0 \\ -1, \text{ if } \theta < 0 \end{cases} \tag{31}$$

Based on Eq. 29 and Eq. 30, it can be concluded that among the geometric parameters, only θ_1 has an effect on the steering resistance of the robot, and it is linearly correlated.

Supplementary Note 5. Comparison with other modular self-reconfigurable robots

We have included a performance comparison chart (Supplementary Fig. 7). This chart benchmarks six key indicators against several existing homogenous freeform modular self-reconfiguring robots. Additionally, we have added a table comparing the variety of tasks that can be executed by our robot swarm against other terrestrial modular self-reconfiguring robots (Table. R1). This comparison highlights the superior capabilities of our current work.

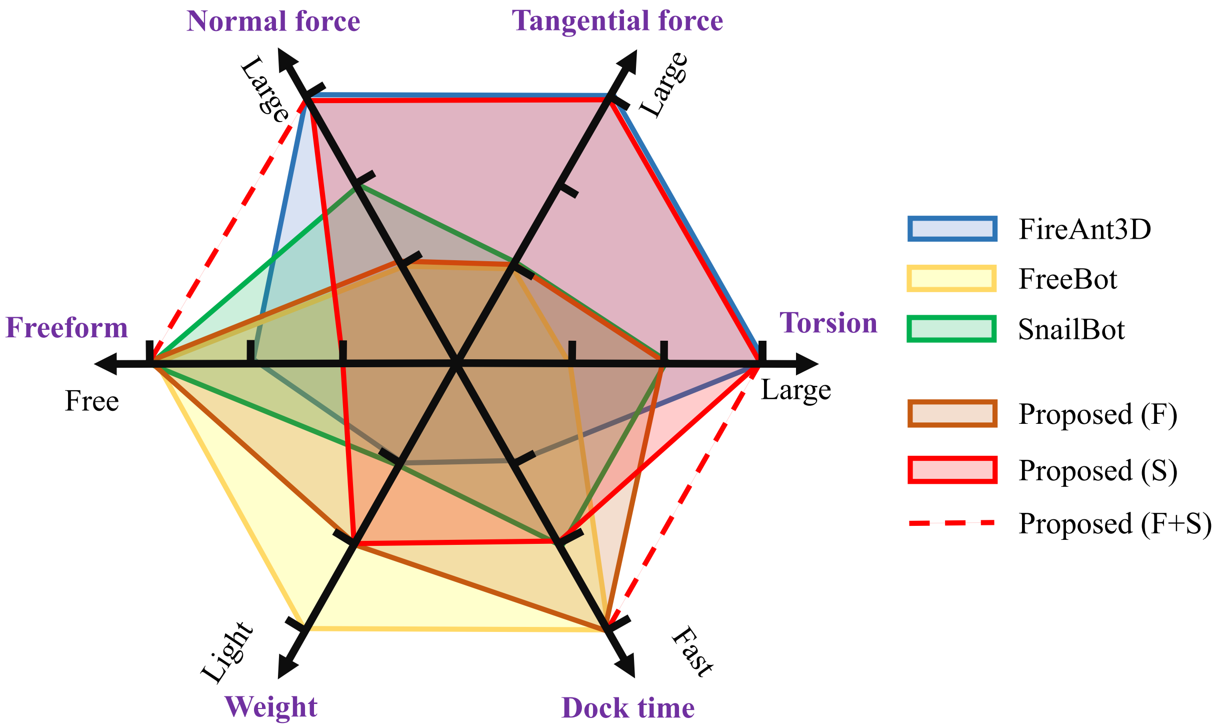

Supplementary Fig. 7. Comparison with other isomorphic modular self-reconfigurable robot. The ticks of each axis from the graph center to the outward are as follows: Freeform: not free, free transition, free connection; normal force: below 50N, above 50N and above 100N.; tangential force: below 50N, above 50N and above 100N., torsion: below 1Nm, above 1Nm and above 5Nm, dock time: above 5s, above 1s and below 1s., weight: above 1kg, above 500g and below 500g. (Proposed (F+S): F means Free mode and S means Strong mode)

Supplementary Table. 1. Task comparison of 3D modular reconfigurable robots.

Robot	Wild mobility	Self-reconfig.	Self-assembly	Locomotion	Manipulation	Flow	Support
This work	✓	✓	✓	✓	✓	✓	✓
SnailBot (ref. 39)		✓	✓		✓	✓	
Mori (ref. 27)		✓	✓	✓	✓		
FreeSN (ref. 40)		✓	✓	✓	✓		✓
SMORES (ref. 22)		✓	✓	✓			
MTRAN-III (ref. 19)		✓		✓	✓		
FreeBOT (ref. 38)		✓	✓	✓	✓	✓	
FireAnt3D (ref. 34)		✓	✓	✓			✓
RoomBot (ref. 23)		✓		✓	✓		✓
M-Blocks (ref. 25)		✓				✓	
SamBot (ref. 20)		✓		✓	✓		
VTT (*1*)		✓		✓			✓

[1] Wild mobility means the ability of single robot's mobility in the wild.
[2] All capabilities of the robots are based on what appears in their related papers.

Supplementary Tables:

Supplementary Table. 2. Specifications of snail robot.

Parameters	Values
Weight	650g
Shell diameter	120mm
Height	122.9mm
Width	120mm
Length	128.3mm
Docking time	≈1s
Maximal connecting No.	4
Continuous operating time	≈30min
Maximal forward speed	0.3body/s
Maximal turning angular velocity	0.6rad/s

Supplementary Table. 3. Specifications of magnets.

Parameters	Values	
	Track magnet	Bottom magnet
Material	N521 neodymium	N52 neodymium
Size	12.7*6.35*3.2(mm)	15*10*6(mm)
Number	16	4
Remanence coefficient	1.47T	1.47T

Supplementary Table. 4. Symbol descriptions.

Symbol	Description
O_B, O_F	body-fixed coordinate frame of the base robot and the follower robot
X_B, Y_B, Z_B	x-axis, y-axis and z-axis of base robot's body frame
X_F, Y_F, Z_F	x-axis, y-axis and z-axis of follower robot's body frame
v_i, ω_i	forward velocity and angular velocity of the robot i in the body frame
v_{track}	linear velocity of the track
ω_R, ω_L, ω_M	angular velocity of the right motor, left motor, and middle motor
$R_{suc.}$	radius of sucker
T	force applied on the DPS
F_n, F_s	external normal force and external shear force
r_i	distance between the single polymeric stalk and the sucker center
τ_z	external torque applied on the z-axis
α	angle between the direction of T and the XY plane of the robot
θ_{tip}, θ_{base}, r_s	parameters of DPS
h, θ_{step}	hight and angle of the step
l	width of the gap
l_1, l_2, l_3, l_4, l_5, l_6 ,R_1, r_1, R	size parameters of a snail robot
θ_1, θ_2, θ_3, θ_4, θ_5, θ_6	angel parameters of a snail robot
d	width of the track
O_1, O_2, O_3, O_4	center of the robot shell (2D)
A_1, A_2, A_3	center of the robot wheel
S_1, S_2, S_3, S_4, S_5	support point
w_1, w_2, w_3	weight coefficient
lb_i, ub_i	lower bound and upper bound of basic sizes
γ	angle between O_1O_2 and the vertical line
γ^*	value of γ when COM and the supporting point are vertically aligned
L	distance between COM and the supporting point in the horizontal direction
G	gravity of the snail robot
μ	friction coefficient between the track and the spherical shell
μ_1	friction coefficient between the track and the ground
F_d	robot's maximum forward driving force
η	transmission efficiency between the motor and the track
τ_m	output torque of the driving motor
i	transmission ratio between the motor and the track drive wheel
F_M	attraction force of the robot's magnets to another robot's spherical shell
F_m	resistance of magnets on the caterpillar to the robot's forward movement
F_1, F_2	tractions of the two tracks
F_{R1}, F_{R2}	resistances of the spherical shell to the tracks
M_μ	steering resistance moment applied on the tracks
M_O	steering torque of the robot along the z axis
M_s	rotation torque of the sucker
F_{q1}, F_{q2}	lateral drag load per unit length of the track
R_t	radius of force applied to the robot by the string
F_{gauge}	measurement value of the force gauge

Supplementary Figures:

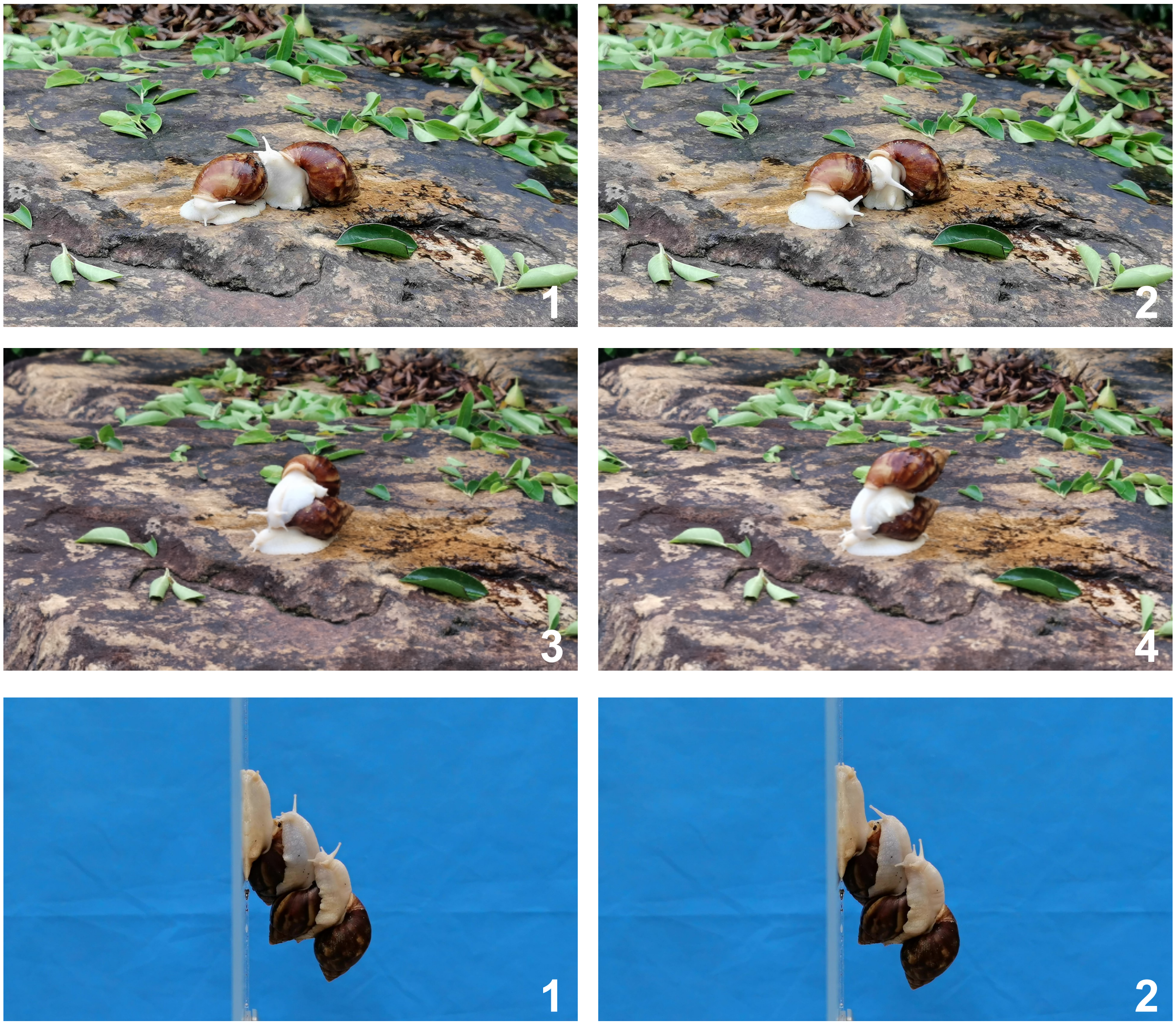

Supplementary Fig. 8. The connection between multiple snails indoors and outdoors.

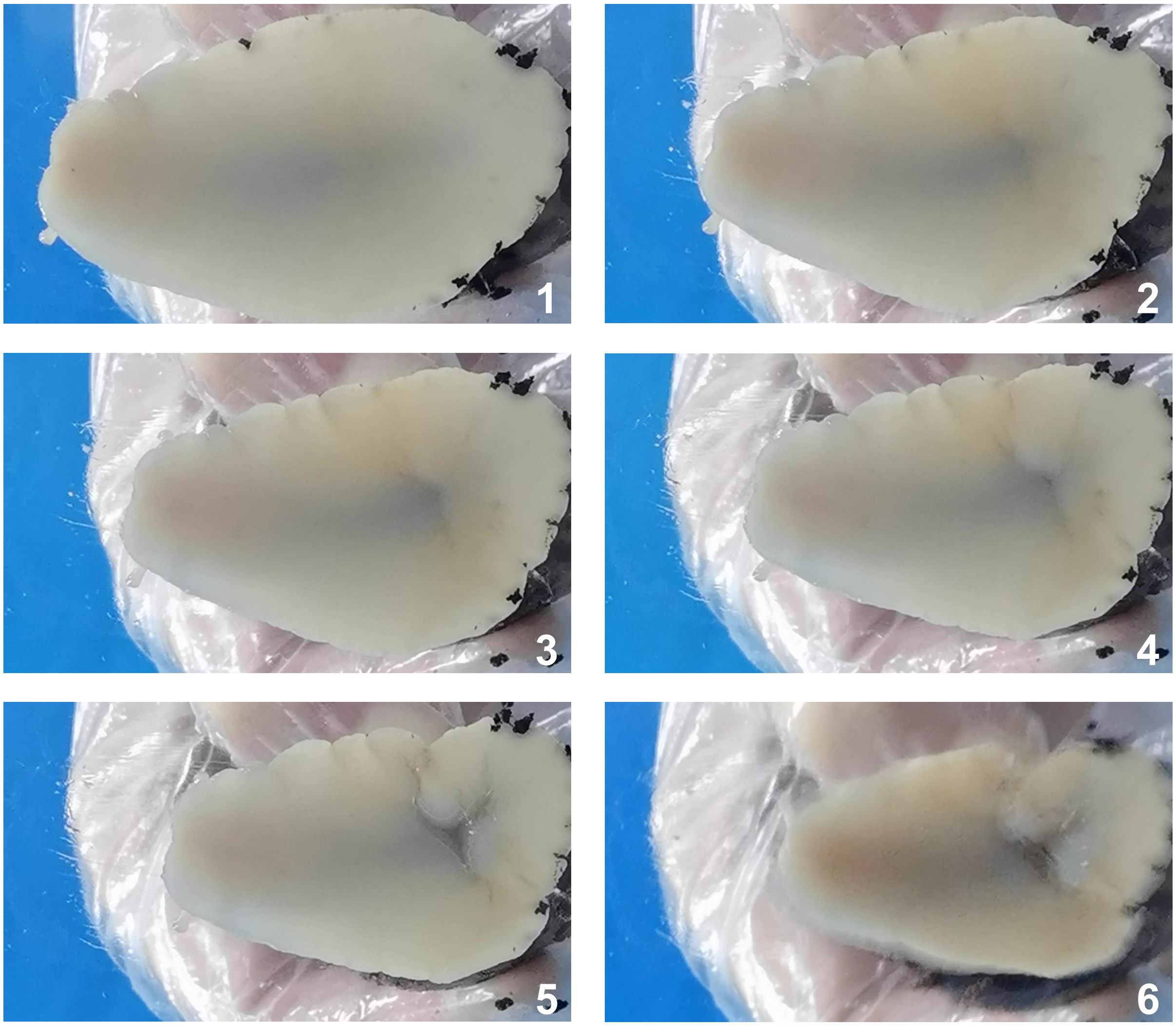

Supplementary Fig. 9. As the external force progressively intensifies, the land snail's connection mechanism shifts its primary reliance from mucus adhesion to suction.

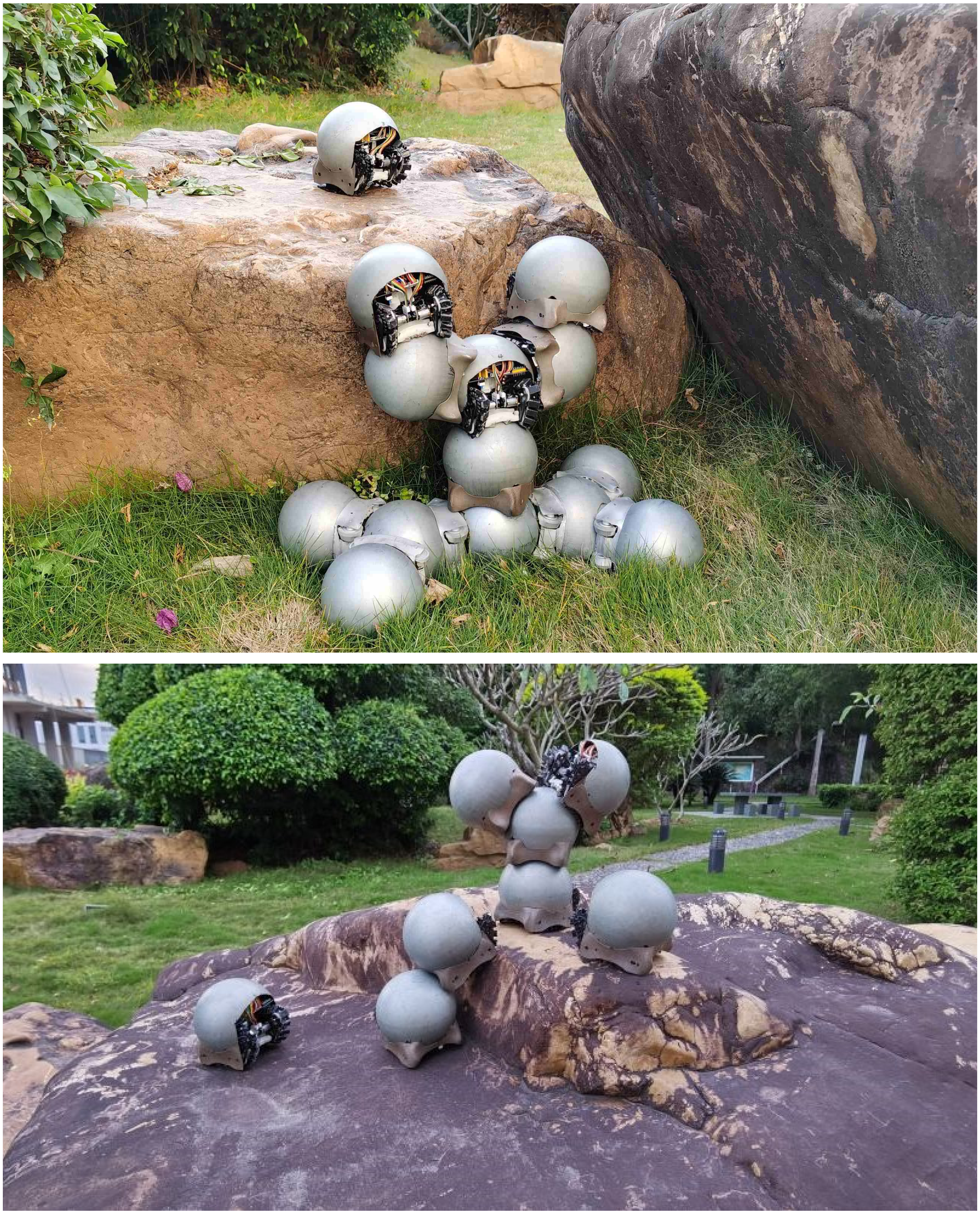

Supplementary Fig. 10. Snail robot swarms in the wild.

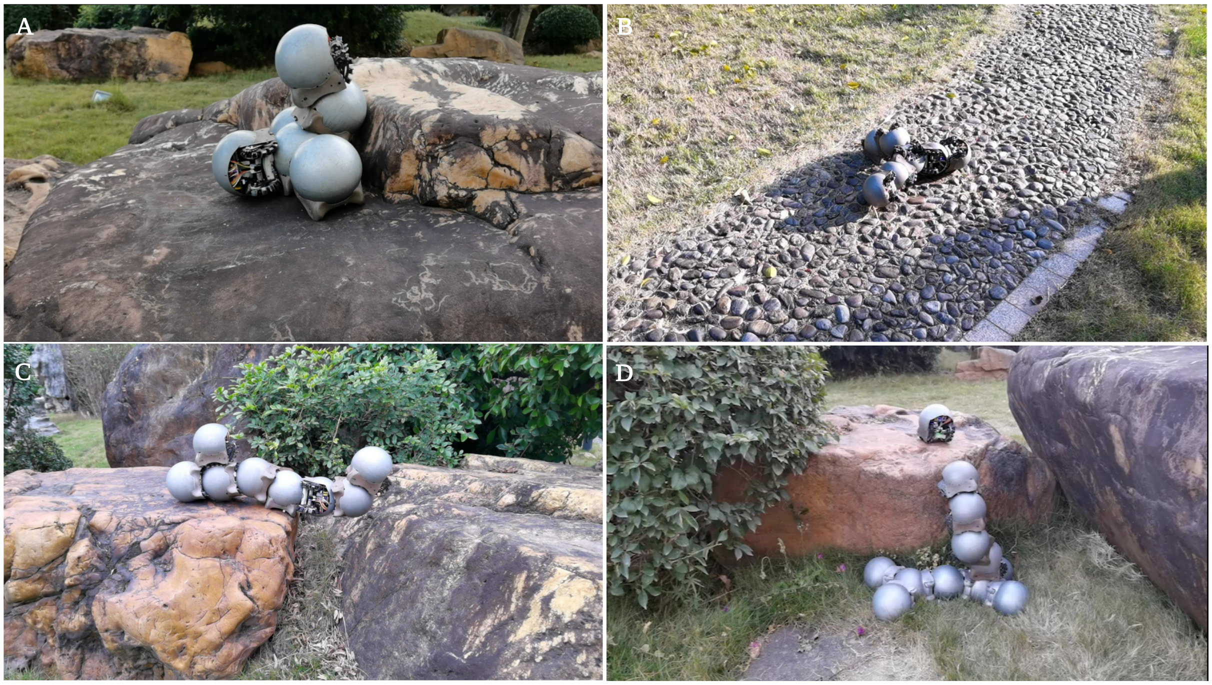

Supplementary Fig. 11. Snail robot swarms outdoor experiments. (**A**) Six snail robots collaborate to climb a natural rock step. (**B**) Seven snail robots form a three-legged system and crawl across the cobblestone road. (**C**) Eight modules collaborate to build a bridge to cross the gully. (**D**) A robotic arm composed of snail robots helps the free module down a boulder.

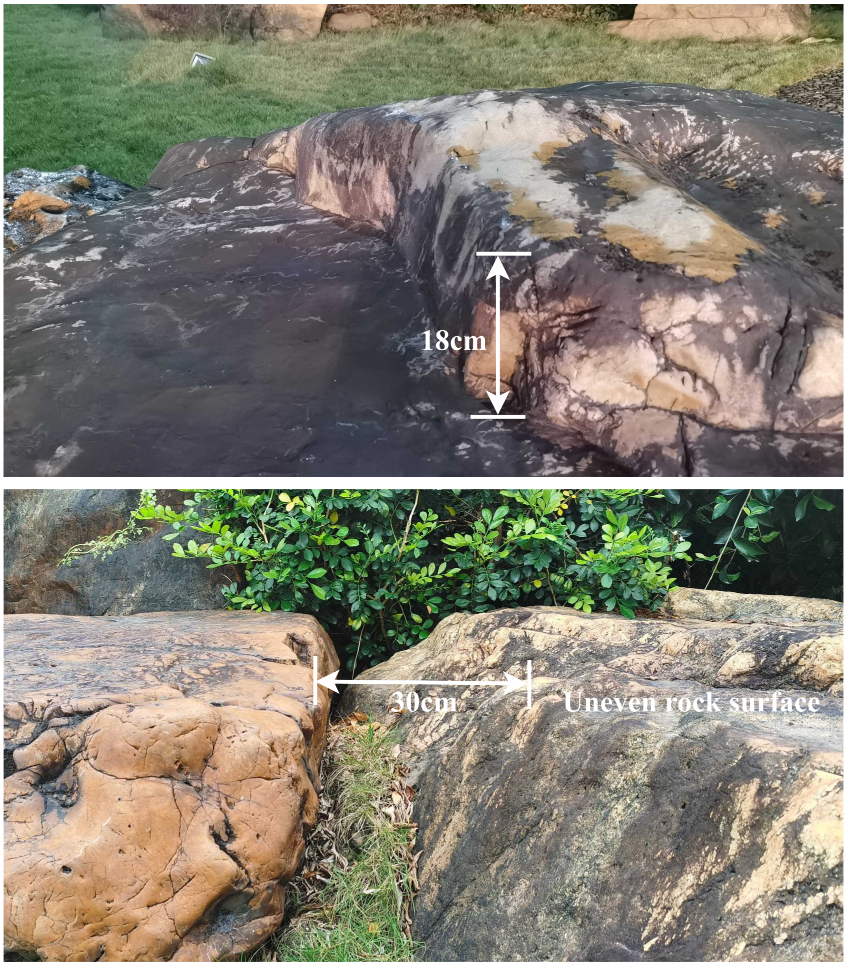

Supplementary Fig. 12. Rock step and gap terrain parameters.

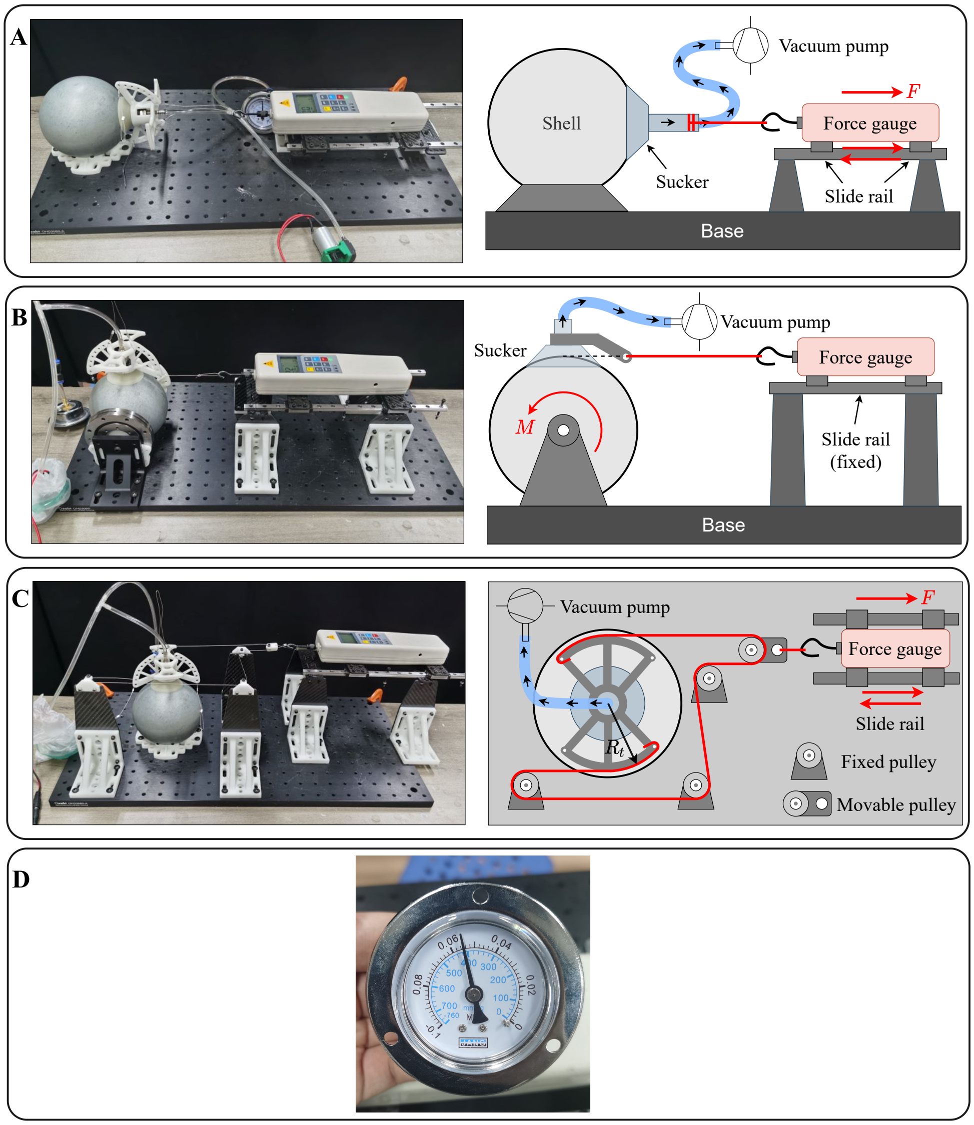

Supplementary Fig. 13. Performance measurement of a sucker. (A) Test the maximum normal force that a vacuum suction cup can withstand: Fix the spherical shell, activate the vacuum pump, and apply a rightward tensile force gauge on the slide rail. The maximum normal force of the active sucker is indicated by the peak value displayed on the force gauge. **(B)** Test the maximum shear force that a vacuum suction cup can withstand: Fix the force gauge and rotate the shell. The maximum shear force of the active sucker is indicated by the peak value displayed on the force gauge. **(C)** Test the maximum torque(z-axis) that a vacuum suction cup can withstand: Fix the spherical shell, activate the vacuum pump, and apply a rightward tensile force gauge on the slide rail. The maximum torque(z-axis) of the sucker is given by $R_t F_{gauge}$. **(D)** The maximum negative pressure value inside the sucker(-0.055Mpa).

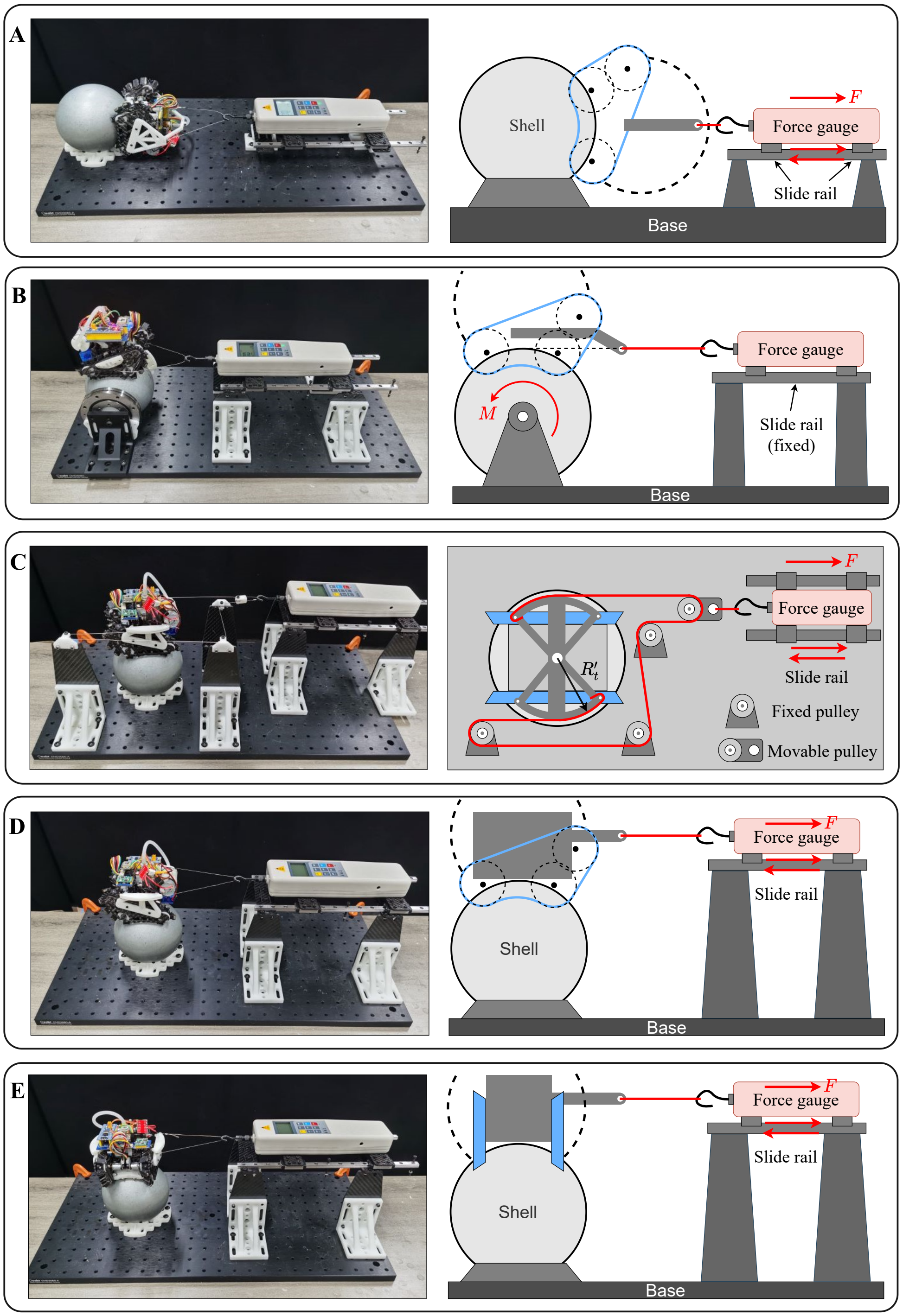

Supplementary Fig. 14. Connector performance measurement of a snail robot. (A) Maximum normal force: Fix the shell and move the force gauge. The maximum normal force is indicated by the peak value displayed on the force gauge. **(B)** Maximum shear force: Fix the force gauge and rotate the shell. The maximum shear force is indicated by the peak value displayed on the force gauge. **(C)** Maximum torque(z-axis): Fix the shell and move the force gauge. The maximum torque(z-axis) of the sucker is given by $R_t'F_{gauge}'$. **(D)** Maximum bending force(x-axis): Move the gauge and the maximum bending force(x-axis) is indicated by the peak value displayed on the force gauge.. **(E)** Maximum bending force(y-axis): Move the gauge and the maximum bending force(y-axis) is indicated by the peak value displayed on the force gauge.

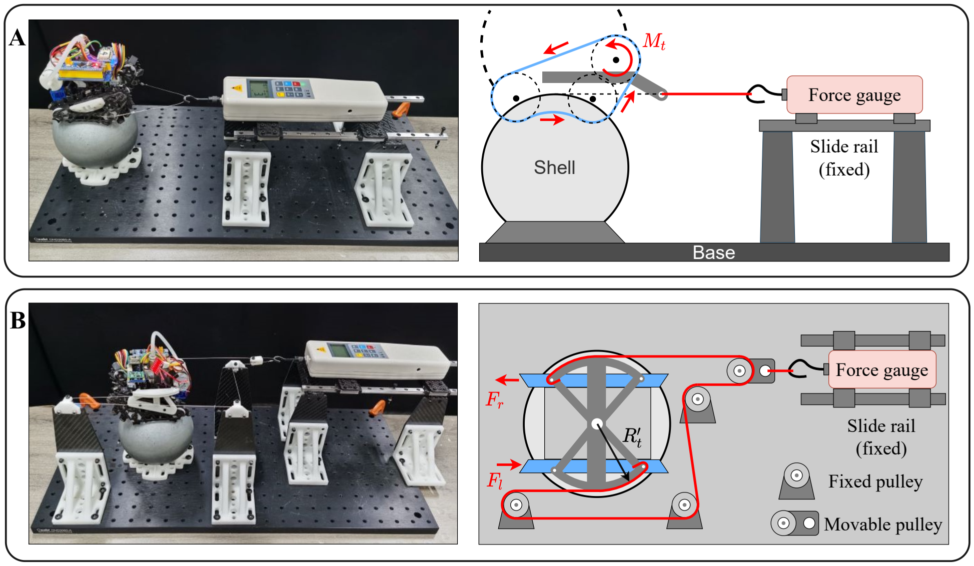

Supplementary Fig. 15. Driving performance measurement of a snail robot. (A) Test the maximum forward driving force when a snail robot is connecting to another robot: Fix the force gauge and the spherical shell. The snail robot moves on its own. The maximum driving force is indicated by the peak value displayed on the force gauge. **(B)** Test the maximum turning torque when a snail robot is connecting to another robot: Fix the force gauge and the spherical shell. Let the robot turns on its own. The maximum turning torque is given by $R_t' F_{gauge}''$.

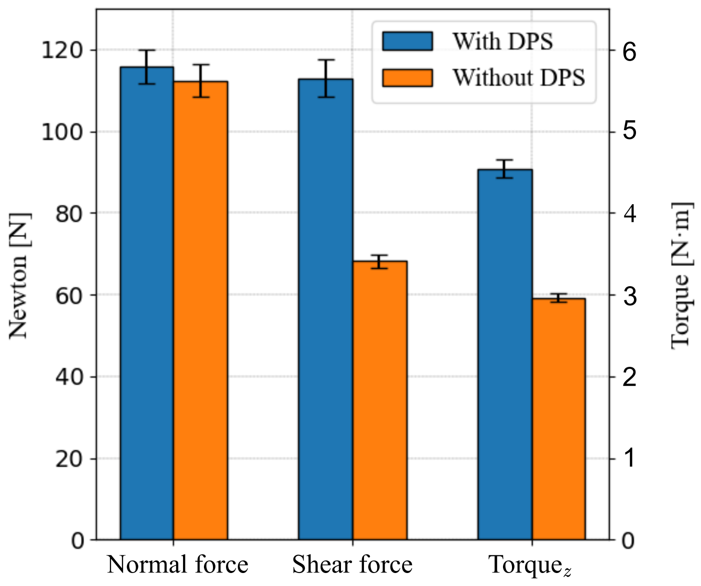

Supplementary Fig. 16. Performance comparison between active suckers with and without DPS.

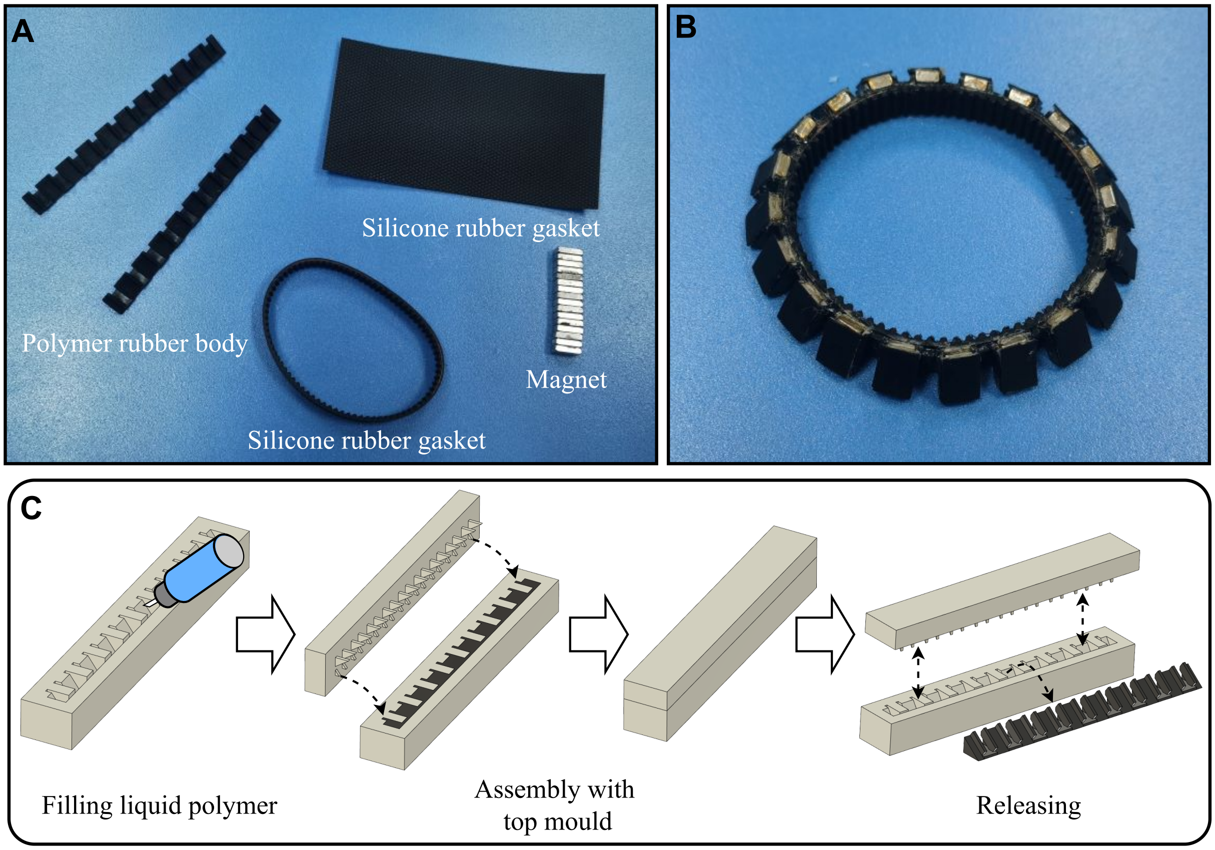

Supplementary Fig. 17. Fabrication of the magnetic track. **(A)** Elements of a magnetic track. **(B)** The finished magnetic track. **(C)** Moding/casting the polymer rubber body.

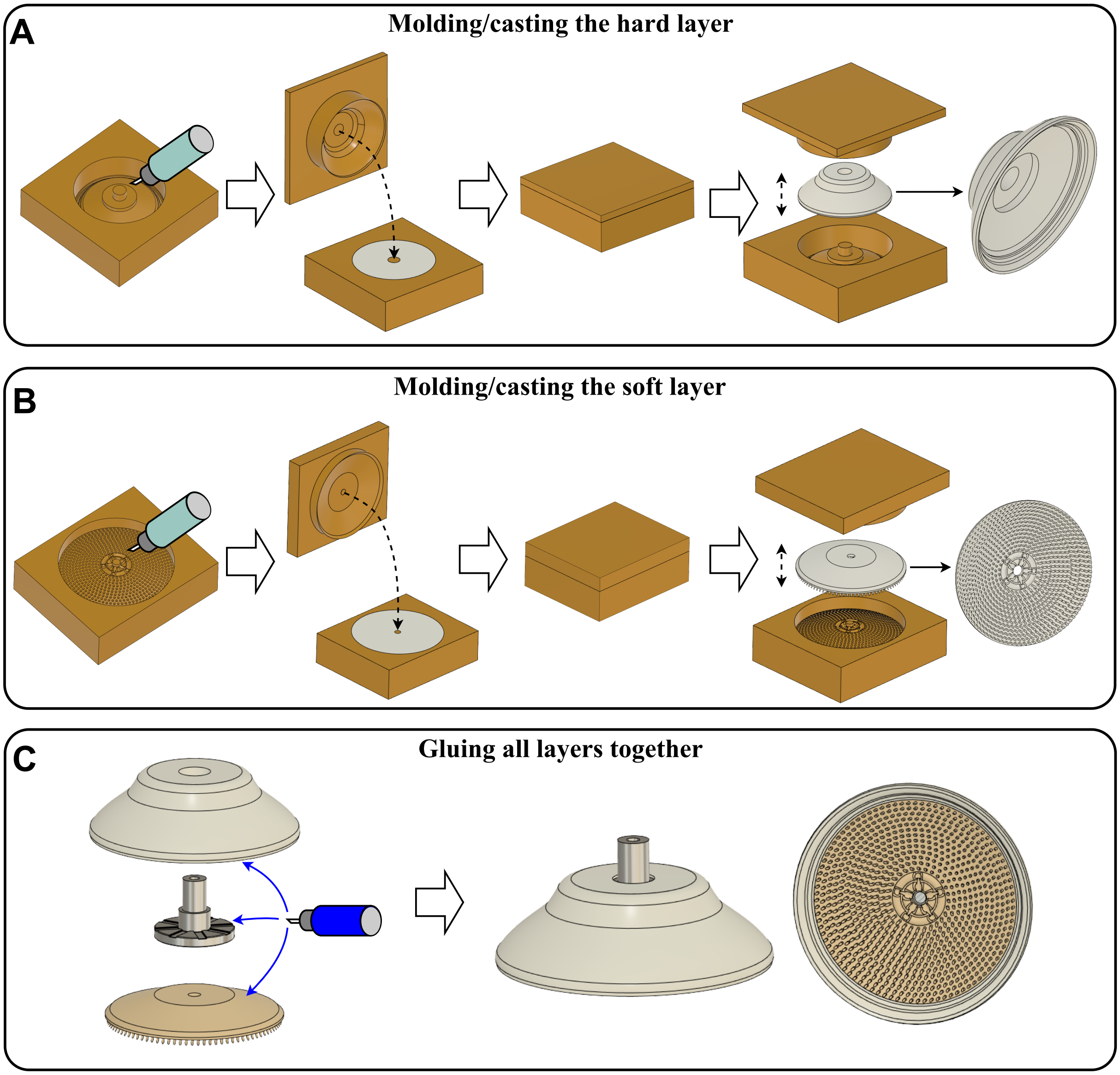

Supplementary Fig. 18. Fabrication of the active sucker with DPS. (A) Molding/casting the hard layer with hard silicone rubber. **(B)** Molding/casting the hard layer with soft silicone rubber. **(C)** Gluing all layers together.

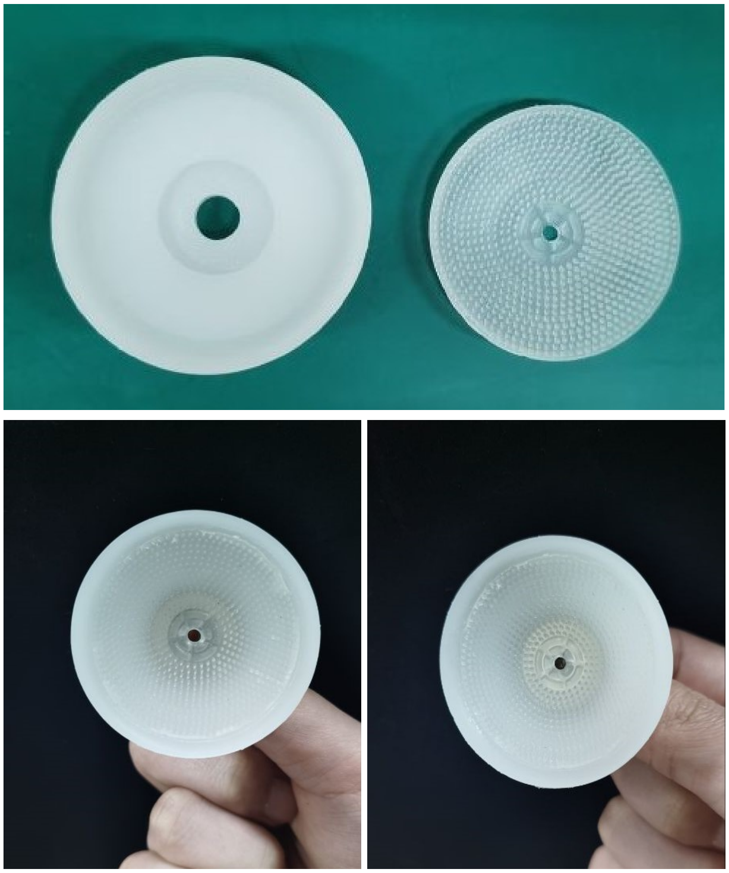

Supplementary Fig. 19. Sucker's two layers and the final prototype.

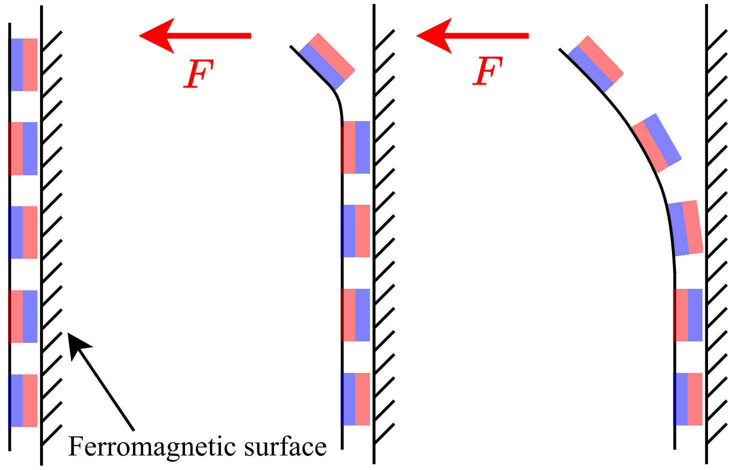

Supplementary Fig. 20. Illustration of the peel-off effect of a magnetic track.

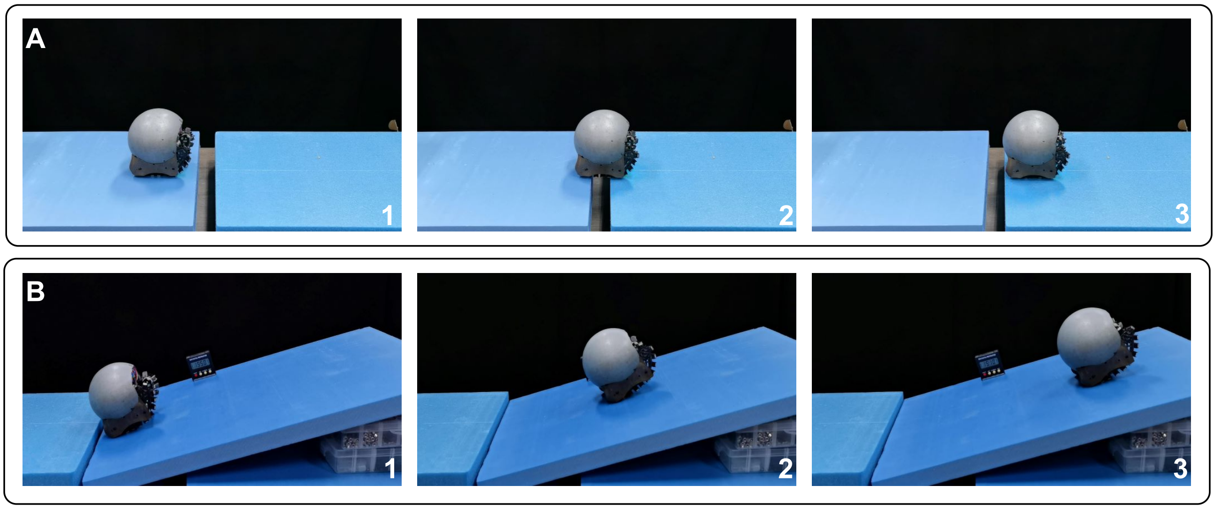

Supplementary Fig. 21. Obstacle-crossing performance test of a single robot. (A) When moving directly towards a gap, a single robot can pass through gaps with a maximum width of 3.7 cm; **(B)** The maximum climbing angle for the robot's forward movement is approximately 15 degrees.

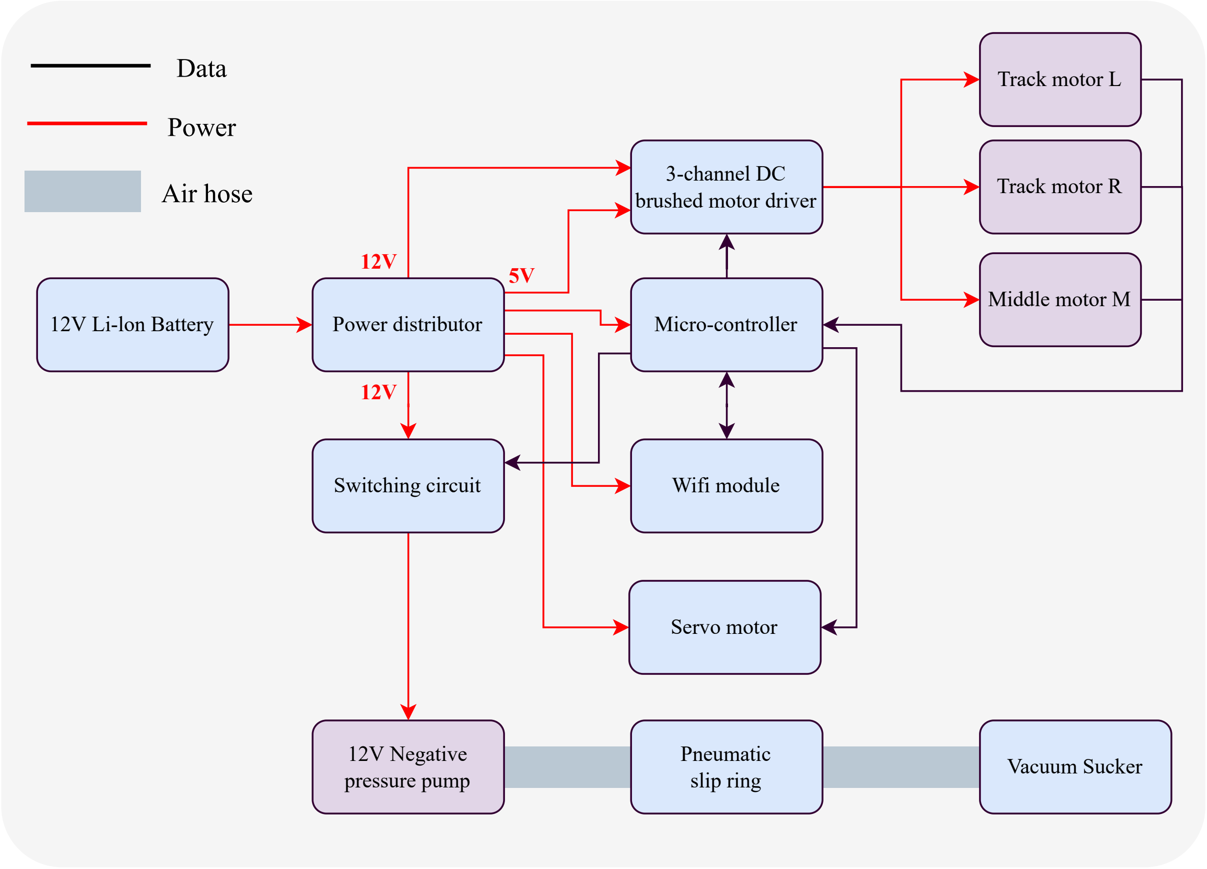

Supplementary Fig. 22. Snail robot's electronic and pneumatic architecture.

Supplementary References

1. E. Park, J. Bae, S. Park, J. Kim, M. Yim, T. Seo, Reconfiguration solution of a variable topology truss: Design and experiment, *IEEE Robotics and Automation Letters* **5**, 1939–1945 (2020).
